# *Mycobacterium microti* Interferes with Bovine Tuberculosis Surveillance

**DOI:** 10.3390/microorganisms8121850

**Published:** 2020-11-24

**Authors:** Lorraine Michelet, Krystel de Cruz, Jennifer Tambosco, Sylvie Hénault, Maria Laura Boschiroli

**Affiliations:** Laboratory for Animal Health, University Paris-Est, Tuberculosis National Reference Laboratory, ANSES, 94701 Maisons-Alfort, France; lorraine.michelet@anses.fr (L.M.); krystel.decruz@anses.fr (K.d.C.); jennifer.tambosco@anses.fr (J.T.); sylvie.henault@anses.fr (S.H.)

**Keywords:** bovine tuberculosis, *Mycobacterium microti*, surveillance, interference

## Abstract

*Mycobacterium microti*, a member of the *Mycobacterium tuberculosis* complex, was originally described as the cause of tuberculosis in wild rodents. However, in the last few years, an increasing number of cases have been reported in wildlife (wild boars and badgers) and livestock (goat and cattle) in the frame of bovine tuberculosis (bTB) surveillance program, demonstrating the risk of interference with bTB diagnosis in France. In 2019, we detected four cattle infected with *M.* *microti*, from three different herds in three different distant regions. For all these cases, ante-mortem diagnosis by the skin test (single intradermal comparative cervical tuberculin (SICCT)) was positive. Confirmation of *M.* *microti* infection was based on molecular tests, i.e., specific real-time PCR and spoligotyping. These results highlight a non-negligible risk of interference in the bTB diagnosis system and raise concern about the reliability of diagnostic tests used for bTB surveillance. The use of highly specific tests, like the interferon gamma test (IFN-γ) employed in France or new synthetic specific tuberculins for skin testing could alternatively be used to accurately identify *M.* *bovis* (or *Mycobacterium caprae*) infection at ante-mortem examination. At post-mortem diagnosis, the use of specific molecular tools should be considered to accurately distinguish pathogens within the MTBC and to avoid misleading bTB diagnosis.

## 1. Introduction

Bovine tuberculosis (bTB) mainly caused by *Mycobacterium bovis* is a transmissible notifiable disease whose prevalence in cattle herds in Europe has been increasing despite long lasting and costly control campaigns. In France—declared officially bTB free in 2001 by the EU—*M. bovis* infection is recurrent at a regional level in cattle and wildlife. The surveillance system in France is based on ante-mortem screening (single intradermal comparative cervical tuberculin (SICCT) and Interferon gamma test (IFN-γ)) in cattle [1]. The periodicity of bTB screening in each region is determined on epidemiological indicators and varies from an annual regime in endemic regions to the absence of any ante-mortem screening in long lasting bTB free zones. In 2019, 867,924 animals from 16,411 TB free herds were skin tested, of which 3705 (0.4%) coming from at least 1738 (10.6%) herds reacted to the skin test (French General Directorate for Food (DGAl) data). Diagnosis of bTB, as recommended by the EU, is based on bacteriology, histology and in France also by PCR. Only 5.8% (215/3705) of the skin test-positive animals were found infected by laboratory analyses, whilst only 5.3% of the reactive herds (92/1738) were confirmed infected (DGAl data). The lack of sensitivity and specificity of ante-mortem tests is of key importance in the final stages of national bovine TB eradication campaigns such as in France.

Since 2010, France has also implemented a surveillance system in wildlife called Sylvatub [2], whose main goal is to detect and monitor *M. bovis* infection in badgers, wild boars and deer through a combination of passive and active surveillance protocols adapted to the estimated risk level in each area of the country.

*Mycobacterium microti*, a member of the *Mycobacterium tuberculosis* complex (MTBC), is the causative agent of tuberculosis in voles. However, infection by this bacterium was described in a large number of other animal species [3]. Recently, an increasing number of *M. microti* infection cases was identified in France through Sylvatub [2], highlighting the potential interference in the surveillance and diagnosis of bTB [4]. Moreover, in France, *M. microti* was also identified in farmed animals, i.e., a goat [5] and a cow [6].

**Figure 1:**
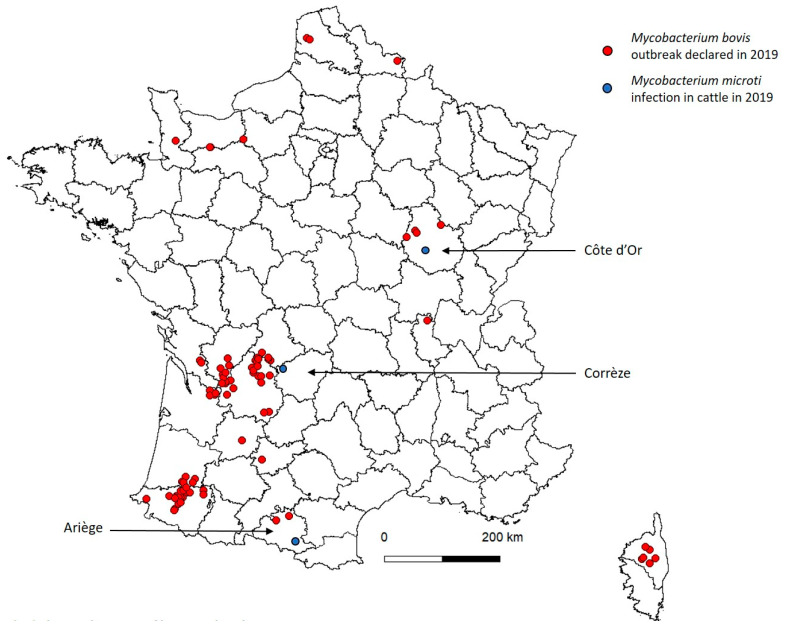
Location of *M. bovis* outbreak declared in 2019 and *M. microti* infection cases in France.

We present here three new cases of *M. microti* infection in four cattle detected successively between December 2018 and July 2019 during bTB surveillance in three regions in France with different bTB epidemiological contexts (Figure 1). These new cases underline the risk of interference of *M. microti* in the diagnosis of bTB and suggest that this infection may be more frequent than previously thought in cattle.

## 2. Materials and Methods

### 2.1. Ethical Statement

BTB is a notifiable disease for which there are control and surveillance campaigns in France. Official methods for diagnosis of this disease are culture, PCR and histopathology. Therefore, all the samples included in this study are issued from animals analyzed within an official context. No purpose killing of animals was performed for this study. All samplings were in complete agreement with national and European regulations. No ethical approval was necessary.

### 2.2. Ante-Mortem Tests

Veterinarians performed SICCT on cattle, as part of the usual official screening program (in compliance with Council Directive 64/432/EEC). Bovine and avian purified protein derivative (PPD) (Zoetis, Louvain La Neuve, Belgium)were injected intradermally and separately in the mid-cervical region, after the measurement of skin-fold thickness at day 0 (thickness at the point of bovine PPD injection: B0; thickness at the point of avian PPD injection: A0). In France, bovine and avian PPD are used at a concentration of 25,000 international units (IU)/mL. The 72 h skin-fold thickness at the avian PPD injection (A3) and bovine PPD (B3) injection site were measured. Differences of skin-fold thickness at injection points were interpreted according to Table 1.

### 2.3. Post-Mortem Diagnosis

Animals were examined because they presented non-negative skin test results and were slaughtered for diagnostic purposes. Tracheobronchial (TB), retropharyngeal (RP), mediastinal (MD) and mesenteric (MS) lymph nodes were sampled on each animal and submitted to the diagnostic tests implemented in France.

Briefly, histopathology was based on Hematoxylin-Eosine and Ziehl Neelsen staining. Bacterial culture is performed following the protocol established by the French National Reference Laboratory (NRL) (NF U 47–104) for isolation of *M. bovis*. Two to 5 g of sampled tissues were crushed with a 4% sulfuric acid solution to decontaminate the tissue. After 10 min, the acid was neutralized by adding a 6% sodium hydroxide solution. After decontamination, the supernatant was seeded on two different solid media: Löwenstein-Jensen and Coletsos. All seeded media were incubated at 37 °C +/- 3 °C for three months and examined every two weeks. Any isolated mycobacterial strain is submitted to the NRL for further characterization.

DNA extraction was performed after mechanical lysis using an LSI MagVetTM Universal Isolation Kit (Life Technologies SAS, Villebon-sur-Yvette, France) with a KingFisherTM Flex automate (Thermo Electron LED S.A.S., Saint Herblain, France), following the manufacturer’s instructions. The LSI VetMAXTM MTBC Real-Time PCR kit (Life Technologies SAS, Villebon-sur-Yvette, France), which targets IS6110 [7] was used to identify the MTBC. IS1561′ and Rv1510 (RD4) based PCRs [8] and spoligotyping by Luminex, as described by Zhang et al. [9] using TB-SPOL kits purchased from Beamedex^®^ (Beamedex SAS, Orsay, France) on Bio-PLex 200/Luminex 200^®^ were used to differentiate *M. microti* vs *M. bovis* infections. The presence or absence of the 43 spacer sequences contained in the DR locus is represented in a binary code of 43 entries. Spoligotypes are named according to an agreed international convention (www.mbovis.org) [10].

## 3. Results

### 3.1. Case 1

In December 2018, in Côte d’Or (Burgundy region, Central East France), a beef cattle organic herd (Salers and Charolaise breeds) of 128 cows was tested by SICCT. This herd has been bTB free since its establishment. Three cows had positive results, and 6 other cows of the same heifer lot gave doubtful results (Table 2). All these animals were culled; no visible lesions (VL) were detected on any of them at slaughterhouse inspection. Samples were submitted to post-mortem diagnosis and two cows, a two-year-old Salers (cattle 1) and a two-year-old Charolaise (cattle 6), gave positive results with the first line MTBC PCR, respectively, on the RP and TB lymph nodes. Further molecular characterization at the NRL identified the MTBC bacillus as *M. microti* spoligotype SB0118.

In this region, skin testing is performed annually. Since 2010, the year of the highest number of incident outbreaks in the department (*n* = 48), the number of incident outbreaks has been decreasing every year. The spatial distribution of the outbreaks shows a decrease in the extent of the endemic area between 2015 and 2017. The number of incident outbreaks detected decreased during these three years in Côte-d’Or (13 in 2015, 11 in 2016 and 3 in 2017) [1]. The situation seems to be under control, with a stable number of incident outbreaks in the last years (3 and 4 in 2018 and 2019) (French General Directorate for Food (DGAl) data).

### 3.2. Case 2

In February 2019, in Corrèze (Nouvelle Aquitaine region, South-West France), 68 cattle from a bTB free since its establishment, were tested to the SICCT test. A four-year-old Limousine was doubtful (DB − DA = 3 mm and DB = 3.5 mm). No VL was detected at slaughterhouse inspection. A positive first line PCR result was obtained on the MD lymph node. Further characterization confirmed *M. microti* spoligotype SB0118 at the NRL.

This case is located in a “reinforced prophylaxis zone” (RPZ) established for the 2017–2018 prophylactic campaign. Risk areas are identified based on the outbreaks observed in cattle and wildlife, and are subject to reinforced annual screening [1]. bTB outbreaks have been sporadically detected in Corrèze department since 2010. This case is very close to the border of Dordogne department, a highly endemic area that records the most important number of *M. bovis* outbreaks in the last years [1].

### 3.3. Case 3

In July 2019, in Ariège (Occitanie region, Southern France), a third case was identified during bTB prophylaxis. This cow belonging to a bTB free since its establishment, gave positive results at the SICCT (DB − DA = 9 mm and DB = 15 mm). The cow was a 5-year-old Gasconne. No VL were observed at slaughterhouse inspection. A positive first-line PCR result was obtained on RP and TB lymph nodes. *M. microti* spoligotype SB0112 was confirmed by the NRL.

In this department, the rhythm of skin testing prophylaxis is triennial. The number of outbreaks identified in this department has been decreasing between 2010 (nine outbreaks) and 2015 (three outbreaks). No outbreak was detected between 2016 and 2018, and there were only two in 2019. However, this department is a hotspot of *M. microti* infection in wildlife [4,11]. Indeed, the surveillance system in wildlife allowed us to identify an increasing number of cases in badgers and wild boars in the last years. *M. microti* cases have also been identified in cats, dogs and llamas [3].

The main results on these three cases are summed up in Table 3.

## 4. Discussion

These consecutive cases of *M. microti* infection in cattle highlight a non-negligible risk of interference in the TB diagnosis system. *M. microti* has previously been isolated in skin test reactor cattle in the UK [12]. In France, we previously recorded *M. microti* infection in a goat and in a cow in a region of the Alps Mountains [5,6], demonstrating the risk of infection in livestock. *M. microti* infection in cattle may not be as rare as previously thought. All cattle were infected by *M. microti* presenting the same genotype circulating in the region and identified in wildlife: SB0118 in Nouvelle Aquitaine, and Burgundy, SB0112 in Occitanie [3,11].

These results raise concern about the reliability of diagnostic tests used for bTB surveillance. The use of highly specific ante-mortem tests based on specific antigens such as ESAT6 and CFP10, which are absent in *M. microti*, and already currently used in the IFN-γ test employed in France [13], could be employed to recognize this kind of MTBC cross infection. Such as in their DIVA (Differentiating Infected from Vaccinated Animals) test basis, alternative new synthetic specific tuberculins could also be extremely useful to accurately identify *M. bovis* (or *Mycobacterium caprae*) infection vs *M. microti* infection at ante-mortem examination as they are mainly based on the BCG and *M. microti* absence of ESAT6 and CFP10 antigens [14].

Even though no visible lesions were detected for these cases, the load of *M. microti* bacilli was quite important. The Ct values we observed in PCR, i.e., 27–34, are very similar to those found in *M. bovis* infected animals and confirmed by bacteriology in our lab. This corresponds roughly to 10^4–^10^2^ CFU/g of infected tissue (Boschiroli personal communication). Besides, the immunological response observed at skin testing were important, which could be a response to the bacillus proliferation as if real infection were taking place in the animal. Without molecular diagnosis, as almost systematically for *M. microti* infections, this bacillus could not have been detected and identified [3,5,6,15,16,17]. At post-mortem diagnosis, the use of specific molecular tools should be considered to rapidly distinguish pathogens within the MTBC and to avoid misleading bTB diagnosis. Interestingly, the genotypes of *M. microti* are those found in the same regions beforehand, highlighting the strong clonality of this MTBC member which is the same phenomenon that we also observe for *M. bovis* in France [18].

The source of cattle *M. microti* exposition and infection remains unknown. It could either be by direct contact of infected rodents in the barns or by indirect contact through consumption of contaminated cattle pastures, food or water sources.

Here, we show once again that, although infection by *M. bovis* and by *M*. *microti* are exclusive individually (until present we have never found co-infected wild or livestock animal [3,4,5,6]), the presence of *M. microti* locally does not prevent the presence of bovine tuberculosis both in livestock and in wildlife at a population level. Thus, *M. microti* would not be conferring a strong protection against TB due to *M. bovis*.

## Figures and Tables

**Table 1:** Interpretation of the results of the single intradermal cervical comparative test (SICCT).

Skin-Fold Thickness at the Bovine PPD Injection (DB ^1^ = B3 − B0)	Difference of Skin-Fold Thickness at the Points of Bovine PPD Injection (DB^1^ = B3 − B0) and Avian PPD Injection (DA ^2^ = A3 − A0)	Result of SICCT
DB ^1^ > 2 mm	DB ^1^ − DA ^2^ > 4 mm	Positive
	1 mm ≤ DB ^1^ − DA ^2^ ≤ 4 mm	Doubtful
	DB ^1^ − DA ^2^ < 1 mm	Negative
DB ^1^ ≤ 2 mm		Negative

^1^ DB: Difference of skin-fold thickness at the points of bovine PPD injection, ^2^ DA: Difference of skin-fold thickness at the points of avian PPD injection.

**Table 2:** Description of bovine tuberculosis diagnosis results.

Cattle	DA	DB	DB-DA	Interpretation	MTBC PCR	Molecular Identification
1	1.9	3.7	1.8	Doubtful	Positive (RP LN ^1^)	*M. microti* SB0118
2	0.7	2.8	2.1	Doubtful	Negative	NA ^3^
3	0	2.7	2.7	Doubtful	Negative	NA ^3^
4	1.9	4.9	3	Doubtful	Negative	NA ^3^
5	0.4	9	8.6	Positive	Negative	NA ^3^
6	0.5	4.8	4.3	Positive	Positive (TB NL ^2^)	*M. microti* SB0118
7	1.5	3	1.5	Doubtful	Negative	NA ^3^
8	1.5	3.3	1.8	Doubtful	Negative	NA ^3^
9	0.1	7.3	7.2	Positive	Negative	NA ^3^

^1^ RP LN: Retropharyngeal lymph node, ^2^ TB LN: Tracheobronchial lymph node, ^3^ NA: not applicable.

**Table 3:** Description of *M. microti* cases.

	Case 1a	Case 1b	Case 2	Case 3
Geographic location	Côte d’Or	Côte d’Or	Corrèze	Ariège
Breed	Salers	Charolaise	Limousine	Gasconne
Age	2 years	2 years	4 years	5 years
Skin test result	Doubtful	Positive	Doubtful	Positive
Presence of lesion	NVL	NVL	NVL	NVL
Infected lymph node	RP	TB	MD	RP/TB
First line PCR	Positive (CT 29)	Positive (CT 34)	Positive (CT 32)	Positive (CT 27)
NRL identification	*M. microti* SB0118	*M. microti* SB0118	*M. microti* SB0118	*M. microti* SB0112

## References

[1] Delavenne C., Pandolfi F., Girard S., Réveillaud E., Jabert P., Boschiroli L., Dommergues L., Garapin F., Keck N., Martin F. (2020). Bovine tuberculosis: Results and analysis of the epidemiological status of metropolitan France between 2015 and 2017. Bull. Epid Santé Anim. Alim..

[2] Reveillaud E., Desvaux S., Boschiroli M.L., Hars J., Faure E., Fediaevsky A., Cavalerie L., Chevalier F., Jabert P., Poliak S. (2018). Infection of Wildlife by *Mycobacterium bovis* in France Assessment Through a National Surveillance System, Sylvatub. Front. Vet. Sci..

[3] Michelet L., de Cruz K., Zanella G., Aaziz R., Bulach T., Karoui C., Henault S., Joncour G., Boschiroli M.L. (2015). Infection with *Mycobacterium microti* in animals in France. J. Clin. Microbiol..

[4] Michelet L., de Cruz K., Phalente Y., Karoui C., Henault S., Boschiroli M.L. (2015). *Mycobacterium microti* detection in French wildlife. Vet. Rec..

[5] Michelet L., de Cruz K., Phalente Y., Karoui C., Henault S., Beral M., Boschiroli M.L. (2016). *Mycobacterium microti* Infection in Dairy Goats, France. Emerg. Infect. Dis..

[6] Michelet L., de Cruz K., Karoui C., Henault S., Boschiroli M.L. (2017). *Mycobacterium microti* infection in a cow in France. Vet. Rec..

[7] Michelet L., de Cruz K., Karoui C., Tambosco J., Moyen J.L., Henault S., Boschiroli M.L. (2018). Second line molecular diagnosis for bovine tuberculosis to improve diagnostic schemes. PLoS ONE.

[8] Lesellier S., Boschiroli M.L., Barrat J., Wanke C., Salguero F.J., Garcia-Jimenez W.L., Nunez A., Godinho A., Spiropoulos J., Palmer S. (2019). Detection of live *M. bovis* BCG in tissues and IFN-gamma responses in European badgers (*Meles meles*) vaccinated by oropharyngeal instillation or directly in the ileum. BMC Vet. Res..

[9] Zhang J., Abadia E., Refregier G., Tafaj S., Boschiroli M.L., Guillard B., Andremont A., Ruimy R., Sola C. (2010). *Mycobacterium tuberculosis* complex CRISPR genotyping: Improving efficiency, throughput and discriminative power of ‘spoligotyping’ with new spacers and a microbead-based hybridization assay. J. Med. Microbiol..

[10] Smith N.H., Upton P. (2012). Naming spoligotype patterns for the RD9-deleted lineage of the *Mycobacterium tuberculosis* complex; www.Mbovis.org. Infect. Genet. Evol..

[11] Perez de Val B., Sanz A., Soler M., Allepuz A., Michelet L., Boschiroli M.L., Vidal E. (2019). *Mycobacterium microti* Infection in Free-Ranging Wild Boar, Spain, 2017–2019. Emerg. Infect. Dis..

[12] Jahans K., Palmer S., Inwald J., Brown J., Abayakoon S. (2004). Isolation of *Mycobacterium microti* from a male Charolais-Hereford cross. Vet. Rec..

[13] Faye S., Moyen J.L., Gares H., Benet J.J., Garin-Bastuji B., Boschiroli M.L. (2011). Determination of decisional cut-off values for the optimal diagnosis of bovine tuberculosis with a modified IFNgamma assay (Bovigam(R)) in a low prevalence area in France. Vet. Microbiol..

[14] Srinivasan S., Jones G., Veerasami M., Steinbach S., Holder T., Zewude A., Fromsa A., Ameni G., Easterling L., Bakker D. (2019). A defined antigen skin test for the diagnosis of bovine tuberculosis. Sci. Adv..

[15] Oevermann A., Pfyffer G.E., Zanolari P., Meylan M., Robert N. (2004). Generalized tuberculosis in llamas (*Lama glama*) due to *Mycobacterium microti*. J. Clin. Microbiol..

[16] Palgrave C.J., Benato L., Eatwell K., Laurenson I.F., Smith N.H. (2012). *Mycobacterium microti* infection in two meerkats (*Suricata suricatta*). J. Comp. Pathol..

[17] Rufenacht S., Bogli-Stuber K., Bodmer T., Jaunin V.F., Jmaa D.C., Gunn-Moore D.A. (2011). *Mycobacterium microti* infection in the cat: A case report, literature review and recent clinical experience. J. Feline Med. Surg..

[18] Hauer A., De Cruz K., Cochard T., Godreuil S., Karoui C., Henault S., Bulach T., Banuls A.L., Biet F., Boschiroli M.L. (2015). Genetic evolution of *Mycobacterium bovis* causing tuberculosis in livestock and wildlife in France since 1978. PLoS ONE.

